# Supplementary material for: Toward a genetic system in the marine cyanobacterium Prochlorococcus
Source: Access Microbiol. 2020 Feb 19;2(4):acmi000107. doi: 10.1099/acmi.0.000107 (PMC7523629; doi:10.1099/acmi.0.000107)
Supplement: Supplementary material 1 [file acmi-2-107-s001.pdf]

SUPPLEMENTAL INFORMATION

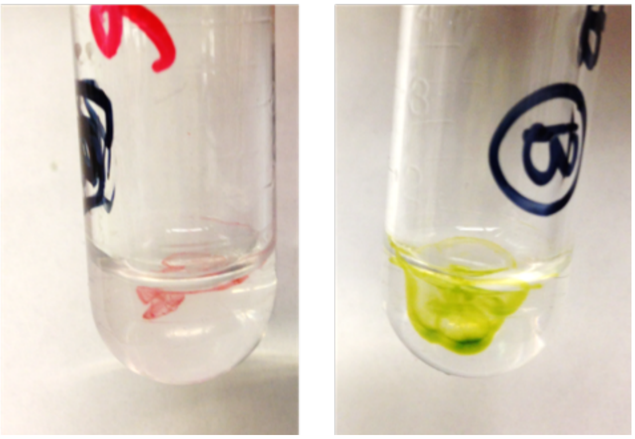

Supplementary Fig. 1. The 'agar stab' mating procedure. The picture shows the 1 mL agar stab following injection with 100 µL mixture of concentrated *E. coli* donor and receiver strain (*Synechococcus* strain WH7803 on the left, *Prochlorococcus* strain MIT9313 on the right). The tubes were placed in the constant light incubator for 24 h to allow mating (see methods for details).

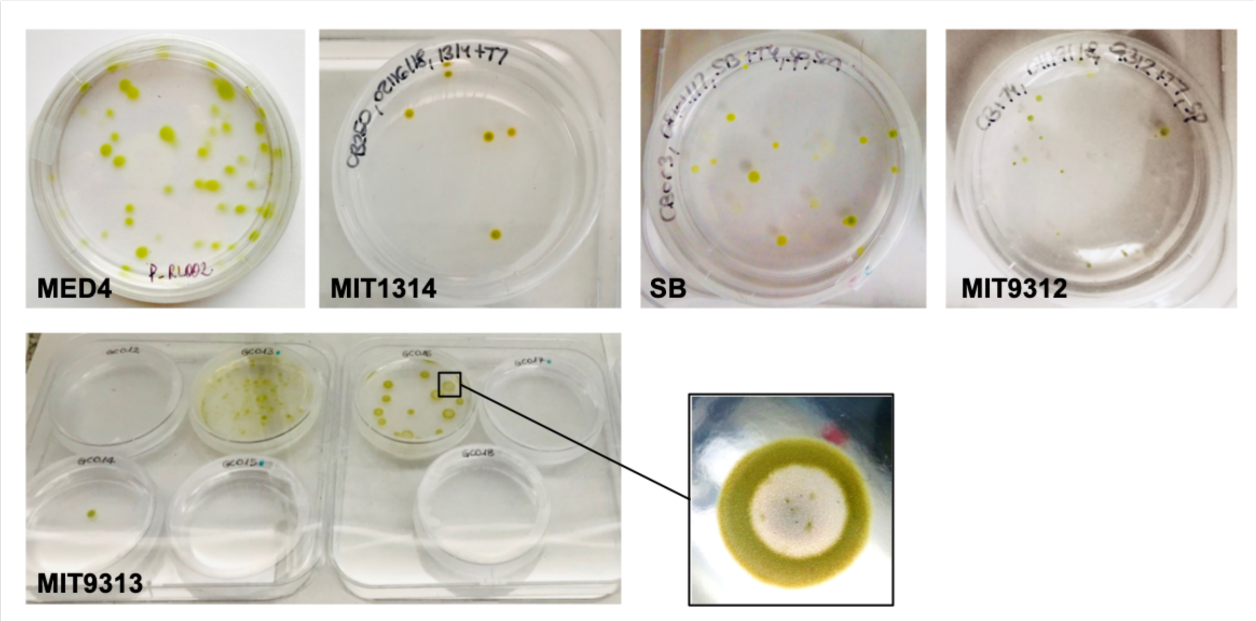

Supplementary Fig. 2. Example of axenic colonies obtained with various strains of *Prochlorococcus*. For all strains, colonies were incubated for at least six weeks. Colonies can appear more or less 'diffused' according to the strain and the incubation time. All colonies were growing within the agar and not at the surface. After eight weeks of growth, colonies sometimes adopt a ring shape, as cells in the center of the colony are dying (see close up picture of a ring-shaped colony).

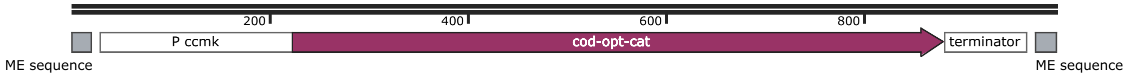


```
  1 ctgtctctta tacacatctc aaccatcatt gatgcactaa ctaatttcga taagtattga
 61 catatcaata gtagcaaggg tgataagttt aacttatcaa tgatagaaaa aacattaata
121 gagtatttag taaaaaatgc ttgacttatc agtacgttat ggaccattct tcggattgaa
181 cattccacat ttagtaatta gtaggcaatg gctacagaaa caatggaaaa aaaaattact
241 ggatatacta ctgttgatat ttctcaatgg catagaaaag aacattttga agcttttcaa
301 tctgttgctc aatgtactta taatcaaact gttcaattag atattactgc tttttaaaa
361 actgttaaaa aaaataaaca taaattttat cctgctttta ttcatatttt agctagatta
421 atgaatgctc atcctgaatt tagaatggct atgaaagatg gagaattagt tatttgggat
481 tctgttcatc cttgttatac tgtttttcat gaacaaactg aaactttttc ttctttatgg
541 tctgaatatc atgatgattt tagacaattt ttacatattt attctcaaga tgttgcttgt
601 tatggagaaa atttagctta ttttcctaaa ggatttattg aaaatatgtt ttttgtttct
661 gctaatcctt gggtttcttt tacttctttt gatttaaatg ttgctaatat ggataatttt
721 tttgctcctg tttttactat gggaaaatat tatactcaag gagataaagt tttaatgcct
781 ttagctattc aagttcatca tgctgtttgt gatggatttc atgttggaag aatgttaaat
841 gaattacaac aatattgtga tgaatggcaa ggaggagctt aagacaattg tctaattaat
901 tgcggaccct agaggtcccc tttttattt taaaaatttt ttcacaaaac ggtttacaag
961 cataaaatct ctgaagatgt gtataagaga cag
```

Supplementary File 1. Transposome T5 map and sequence; ‘cod-opt-cat’: codon-optimized chloramphenicol resistance; ‘ME sequence’: transposase Tn5 Mosaic End recognition sequence.

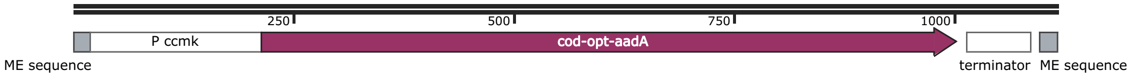


```
   1 ctgtctctta tacacatctt tgatgcacta actaatttcg ataagtattg acatatcaat
  61 agtagcaagg gtgataagtt taacttatca atgatagaaa aaacattaat agagtattta
 121 gtaaaaaatg cttgacttat cagtacgtta tggaccattc ttcggattga acattccaca
 181 tttagtaatt agtaggcaat ggctacagaa acaatgaggg aagccgtaat cgctgaggta
 241 tcgacccaac tttccgaagt tgttggagtt atcgaaagac atttagaacc cacactgttg
 301 gcggtgcatc tatatggttc ggctgttgat ggagggttaa aaccccattc tgacatcgac
 361 ctcttggtta ctgtaaccgt tcgattggat gaaaccactc gccgagcatt aattaacgat
 421 cttcttgaaa caagtgcttc tccaggagag agtgaaattt taagagctgt agaagttact
 481 atcgtggtac acgatgacat cattccttgg aggtatcctg ctaaaagaga acttcaattt
 541 ggcgaatggc agagaaatga cattcttgct ggcattttttg aacctgctac tatagatatt
 601 gatttggcaa tccttttaac taaagcacgt gaacattcag tagcattagt tggacctgca
 661 gcagaggagc tattcgatcc tgtgcctgag caagatttat tcgaagcact gaatgagaca
 721 cttactctat ggaatagtcc tccagattgg gccggtgatg aacgcaatgt agttttaaca
 781 ttatctcgaa tttggtattc cgcggttact ggaaaaattg cccccaaaga tgtcgcagca
 841 gactgggcta tggagcgatt accagcacaa tatcagcctg taattttgga agcacgacaa
 901 gcatacctag gtcaagaaga agaccgttta gccagcagag ccgaccaact cgaagagttt
 961 gttcattatg taaaaggaga gattaccaaa gtggttggta agtaagacaa ttgtctaatt
1021 aattgcggac cctagaggtc cccttttta ttttaaaaat tttttcacaa aacggtttac
1081 aagcataaaa tctctgaaga tgtgtataag agacag
```

Supplementary File 2. Transposome T8 map and sequence; 'cod-opt-aadA': codon-optimized streptomycin/spectinomycin resistance; 'ME sequence': transposase Tn5 Mosaic End recognition sequence.
